# Chagas Parasite Detection in Blood Images Using AdaBoost

**DOI:** 10.1155/2015/139681

**Published:** 2015-03-11

**Authors:** Víctor Uc-Cetina, Carlos Brito-Loeza, Hugo Ruiz-Piña

**Affiliations:** ^1^Facultad de Matemáticas, Universidad Autónoma de Yucatán, Anillo Periférico Norte, Tablaje Catastral, 13615 Mérida, YUC, Mexico; ^2^Centro de Investigaciones Regionales Dr. Hideyo Noguchi, Universidad Autónoma de Yucatán, Avenida, Itzáes No. 490 x 59, Colonia Centro, 97000 Mérida, YUC, Mexico

## Abstract

The Chagas disease is a potentially life-threatening illness caused by the protozoan parasite, *Trypanosoma cruzi.* Visual detection of such parasite through microscopic inspection is a tedious and time-consuming task. In this paper, we provide an AdaBoost learning solution to the task of Chagas parasite detection in blood images. We give details of the algorithm and our experimental setup. With this method, we get 100% and 93.25% of sensitivity and specificity, respectively. A ROC comparison with the method most commonly used for the detection of malaria parasites based on support vector machines (SVM) is also provided. Our experimental work shows mainly two things: (1) Chagas parasites can be detected automatically using machine learning methods with high accuracy and (2) AdaBoost + SVM provides better overall detection performance than AdaBoost or SVMs alone. Such results are the best ones known so far for the problem of automatic detection of Chagas parasites through the use of machine learning, computer vision, and image processing methods.

## 1. Introduction

The Chagas disease, also known as American trypanosomiasis, is a potentially life-threatening illness caused by the protozoan parasite,* Trypanosoma cruzi* (*T. cruzi*). According to the World Health Organization [1], it is found mainly in Latin America, where it is mostly transmitted to humans by the faeces of triatomine bugs. More than 25 million people are at risk of the disease and an estimated 10 million people are infected worldwide, mostly in Latin America where Chagas disease is endemic. Approximately 20,000 deaths attributable to Chagas disease occur annually [2].

The Chagas disease presents itself in two phases. The initial, acute phase lasts for about two months after infection. During the acute phase, a high number of parasites circulate in the blood. When the Chagas disease is diagnosed early in this phase and a treatment is initiated, the patient can be cured. During the chronic phase, the parasites are hidden mainly in the heart and digestive muscle. In later years the infection can lead to sudden death or heart failure caused by progressive destruction of the heart muscle.

Some tests can be useful for making a diagnosis, depending on the phase of the disease. According to [3], the most typical tests used for the diagnosis of the Chagas disease are blood culture, chest X-ray echocardiogram, electrocardiogram (ECG), enzyme-linked immunoassay (ELISA), and peripheral blood smears. Up to date, one of the most effective ways of detecting the Chagas disease in its initial phase is through the ELISA test. Another commonly used method is the Chagas Stat-Pak rapid immunochromatographic test [4], which provides a performance comparable to that obtained with ELISA.

Screening blood donors for Chagas disease is of much concern in all Latin American countries. Although the World Health Organization (WHO) expert committee and some guidelines recommend a single ELISA test to screen blood donors [5], in some countries, such as Brazil, there is a more restrictive regulation, recommending two simultaneous tests of different techniques [6], performed in parallel. One of the tests that can be performed in parallel is the inspection of peripheral blood smears.

A peripheral blood smear is basically a glass microscope slide coated on one side with a thin layer of venous blood. The slide is stained with a dye, usually Wright's stain, and examined under a microscope. Even though visual detection of the Chagas parasite through microscopic inspection of peripheral blood smears is the most widely used technique for parasitemia determination, it is a time-consuming and laborious process. When the number of blood screenings performed in a laboratory increases, it becomes a problem. To cope with this problem, we introduce an automatic computational method for the detection of Chagas parasites based on machine learning and image processing algorithms. Chagas detection using automatic image analysis is, to the best of our knowledge, not yet studied as it is evidenced by the lack of publications on this topic.

Currently, there is only a couple of papers reporting results on Chagas parasites detection using machine learning methods [8, 9]. In the former, a Gaussian discriminant analysis is implemented and the resulting performance rates are 0.0167 false-negatives, 0.1563 false-positives, 0.8437 true-negatives, and 0.9833 true-positives. In the latter, a *k*-nearest neighbors binary classifier is trained and the performances are 0.98 and 0.80 in sensitivity and specificity terms, respectively. The results reported in this paper using AdaBoost + SVM have a significant improvement over the previous ones.

Apart from these two papers, no other study using machine learning and computer vision methods has been reported for the detection of the Chagas parasite, to the best of our knowledge. However, such kind of techniques have been extensively used for the detection of Malaria parasites [10–16]. All the approaches we reviewed utilize supervised and/or unsupervised learning methods to detect, classify and quantify the number of malaria parasites on blood images. The general process commonly reported can be divided in three stages: (1) segmentation; (2) features extraction; and (3) classification.

In the first stage, the segmentation is obtained through different methods based on image histrogram computations. The second stage is performed through the computation of different features that can be classified in four categories [15]: texture features, color features, geometric features, and features obtained from human expert knowledge. The third and final stage makes the biggest difference among the methods, some applied neural networks [10], some others applied statistical measures [11, 13], bayesian classifiers [12], *k*-means clustering [17], and other methods [14, 16]. Brief summaries of the approaches more relevant for our own work are presented next.

In [10], an image classification system implementing a two-stage tree classifier using back-propogation neural networks is introduced. Such a system identifies malaria parasites present in thin blood smears and classifies them according to their different species. Image features based on color, texture, and the geometry allow the system to perform morphological and threshold selection of possible parasites, distinguishing them from sane erythrocytes and other cells.

Similarly, the study presented in [11] provides a method for quantification and classification of erythrocytes in stained thin blood films infected with malaria parasites. This approach is composed of three main phases. First, there is a preprocessing step, which corrects luminance differences. Second, there is a segmentation step that uses the normalized RGB color space for classifying pixels either as erythrocyte or background. Third, there is a two-step classification process identifies infected erythrocytes and diagnoses the infection stage, using a trained bank of classifiers. An interesting characteristic of this method is that user intervention is allowed when the approach cannot make a proper decision. Automatic identification of infected erythrocytes showed a specificity of 99.7% and a sensitivity of 94%. Meanwhile, the infection stage was determined with an average sensitivity of 78.8% and average specificity of 91.2%.

In [12], the parasite detector uses a Bayesian pixel classifier to mark stained pixels. The class conditional probability density functions of the stained and the nonstained classes are estimated using a nonparametric histogram method. The stained pixels are further processed to extract features such as Hu moments, relative shape measurements, and color for a parasite or nonparasite classifier. Finally a distance weighted *k*-nearest neighbor classifier is trained with the extracted features achieving 74% of sensitivity, 98% of specificity, 88% of positive prediction, and 95% of negative prediction values for the malaria parasite detection.

Another malaria study based on pattern matching and parameter optimization is presented in [13]. In this case, the parasitaemia measurements are carried out by partitioning the uninfected and infected cells using unsupervised machine learning. A comparison of the performance is done with a training-based method which improves the classification rates giving 92% of precision and 95% of recall.

**Figure 1:**
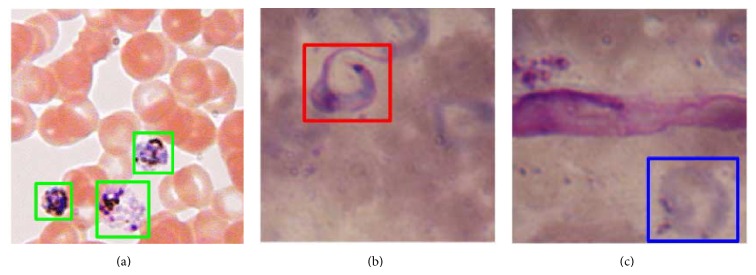
(a) Malaria parasites in early form (inside green squares); (b) Chagas parasite (inside red square); (c) blood cell that looks very similar to a Chagas parasite (inside blue square).

Even when some machine learning methods have been successfully applied to the detection, classification, and quantification of Malaria parasites, these methods cannot be employed in a straightforward way to detect Chagas parasites for one powerful reason: their morphology is different. Malaria parasites have a ring shape (Figure 1(a)) while Chagas parasites have a curved shape, similar to a shrimp (Figure 1(b)). This difference in their morphology prevents us from using exactly the same features during the very important stage of features selection.

In addition to detection algorithms, some authors have developed complete automated systems. In [18], an automated system to identify and analyze parasite species on thick blood films by image analysis techniques is presented. The system comprises two main components: (1) image acquisition unit and (2) image analysis module. The authors have developed an image acquisition system that can be easily mounted on most conventional light microscopes. It automatically controls the movement of microscope stage in 3-directional planes. The vertical adjustment (focusing) can be made in a nanometer range (7–9 nm). Images are acquired with a digital camera that is installed at the top of the microscope. The captured images are analyzed by an image analysis software which utilizes computer vision algorithms to detect and identify malaria parasites.

Other works related to the application of pattern recognition methods for images taken with microscopes are those used for the detection of special types of cells such as cancerous cells [19] and cervical cells [20]. In the first work the authors used the shape, size, and texture of the cells to perform a classification, meanwhile in the second work a *k*-means clustering algorithm for designing binary tree classifiers is used, along with the Bhattacharyya distance metric.

In this paper, we present a comparison of two of the most robust algorithms for binary classification. Both methods have been extensively studied and tested with difficult pattern recognition problems on images such as face detection [21, 22]. We provide an AdaBoost and a SVM learning solution to the task of Chagas parasite detection in blood images. Our AdaBoost solution includes the definition of a new set of Haar-like features specially designed for learning the Chagas parasite's morphology pattern. We give details of the algorithms and our experimental setup. With the best resulting method, AdaBoost, we get 100% and 93.25% of sensitivity and specificity, respectively. Our experimental work shows mainly two things: (1) Chagas parasites can be detected automatically using machine learning methods and (2) AdaBoost + SVM provides better detection performance than plain AdaBoost or SVMs.

In the next section, we provide a brief review of the two compared machine learning methods: AdaBoost and SVM.  Section 3 describes the four stages required to analyze the images looking for subwindows containing a Chagas parasite. The experimental work is detailed in Section 4, which contains the experimental methodology we followed and the experimental results. In Section 5, we discuss the results and point out the issues that need to be addressed if the system is to be established and deployed in reality. Finally, our conclusions are explained in Section 6.

## 2. AdaBoost and SVM

AdaBoost is the short for Adaptive Boosting which is currently the most used boosting method. The goal of boosting is to improve the accuracy of any given learning algorithm. Boosting creates an ensemble of classifiers by training and adding one component classifier at a time. Each new classifier is trained using a different subset of examples. The new training subset contains examples that are incorrectly classified by the current ensemble. By doing such an iterative selection of difficult examples, boosting methods improve the accuracy of any supervised machine learning algorithm. Although each component classifier has an accuracy just greater than average, the joint decision rule of the ensemble has a high accuracy with all previously selected training examples [23].

### 2.1. Boosted Cascade of Simple Features

**Figure 2:**
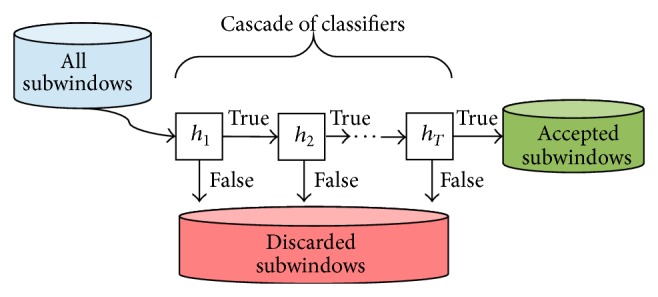
The detection cascade. Classifiers with increasing complexity are arranged in a cascade scheme to allow background regions of the image to be quickly discarded while spending more computing resources on promising regions.

In 2001, Viola and Jones [7] introduced an extremely rapid approach for visual object detection motivated by the task of face detection. Such an approach has as its central component an algorithm based on the standard AdaBoost learning method. In addition to the use of boosting, two are the characteristics that make this approach very fast and efficient. First, the use of the so-called integral image allows the features used by the detector to be computed very quickly. Second, a method for combining the increasingly more complex classifiers in a cascade, as illustrated in Figure 2, allows background regions of the image to be quickly discarded while spending more computing resources on promising regions.

The algorithm introduced by Viola and Jones is presented next.

(i) Given example images (*x*_1_, *y*_1_),…, (*x*_*n*_, *y*_*n*_) where *y*_*i*_ = 0,1 for negative and positive examples, respectively.
(ii) Initialize weights *w*_1,*i*_ = 1/2*m*, 1/2*l* for *y*_*i*_ = 0,1 respectively, where *m* and *l* are the number of negatives and positives, respectively.
(iii) For *t* = 1,…, *T*:
  (1) Normalize the weights,
    (1) $$\begin{array}{c} w_{t,i}⟵\frac{w_{t,i}}{\sum_{j=1}^{n}w_{t,j}} \end{array}$$
    so that *w*_*t*_ is a probability distribution.
  (2) For each feature, *j*, train a classifier *h*_*j*_ which is restricted to using a single feature. The error is evaluated with respect to *w*_*t*_, *ϵ*_*j*_ = ∑_*i*_ *w*_*i*_ | *h*_*j*_(*x*_*i*_) − *y*_*i*_|.
  (3) Choose the classifier, *h*_*t*_, with the lowest error *ϵ*_*t*_.
  (4) Update the weights:
    (2) $$\begin{array}{c} w_{t+1,i}=w_{t,i}\beta_{t}^{1-e_{i}}, \end{array}$$
    where *e*_*i*_ = 0 if, for example, *x*_*i*_ is classified correctly, *e*_*i*_ = 1 otherwise, and *β*_*t*_ = *ϵ*_*t*_/(1 − *ϵ*_*t*_).
(iv) The final strong classifier is
  (3) $$\begin{array}{c} h\left(x\right)=\left\{\begin{array}{cc} 1 & \overset{T}{\underset{t=1}{\sum }}\alpha_{t}h_{t}\left(x\right)\geq \frac{1}{2}\overset{T}{\underset{t=1}{\sum }}\alpha_{t},\\ 0 & \text{otherwise}, \end{array}\right. \end{array}$$
  where *α*_*t*_ = log⁡⁡(1/*β*_*t*_).

### 2.2. Support Vector Machines

A support vector machine (SVM) is a learning tool that originated in modern statistical learning theory [24]. In recent years, SVM learning has found a wide range of real-world applications, including handwritten digit recognition, object recognition, speaker identification, face detection in images, and text categorization. The formulation of SVM learning is based on the principle of structural risk minimization. SVM tends to perform well when applied to data outside the training set and it has been reported that SVM-based approaches are able to significantly outperform competing methods in many applications. SVM achieves this by focusing on the training examples that are most difficult to classify. These training examples are called the support vectors.

## 3. Chagas Parasites Detection

**Figure 3:**
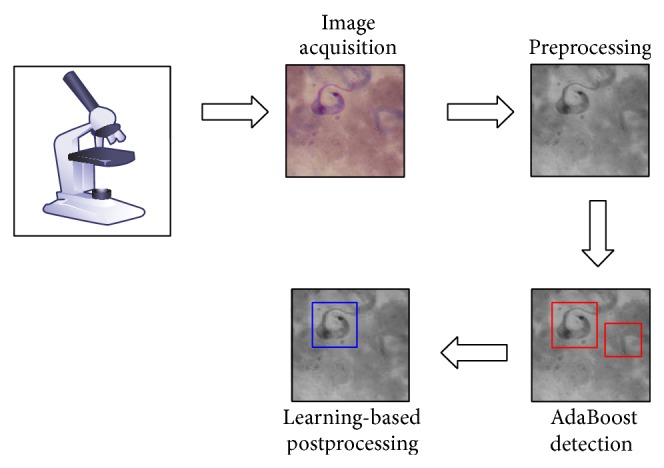
The process of parasite detection consists of four stages: (1) image adquisition via microscope; (2) image conversion from RGB color to grayscale format; (3) possible parasites detection using AdaBoost; and (4) amount of DNA pixels used to further discard false positives.

The whole process of Chagas detection is divided into four stages, as illustrated in Figure 3. This process has been specially designed to allow the automatization of the diagnosis as much as possible. The robustness of our methodology relies on the four modular stages that can be implemented as separated programming classes, where even parallel programming, that is, using Graphics Processing Units (GPUs), can also be employed to reduce the time of detection [25]. This methodology is generic enough to be applied in other object detection systems, particularly from the biology domain where samples observed through a microscope are stained.

The first step consists in obtaining a digital image using a camera and a microscope. Once we have the image in RGB format, in a second step we convert it to a grayscale image to reduce the amount of information that the AdaBoost algorithm needs to process. In the third stage, the grayscale image is scanned using our previously trained AdaBoost classifier in order to detect subwindows of pixels containing possible parasites. Finally, in the fourth step, we use the green component of the original RGB image to extract some three features related to the number of pixels representing a high DNA (deoxyribonucleic acid) content. Based on the amount of DNA it is possible to discard false parasites. This last stage is implemented with a SVM and it is very important because it allows us to include, as part of the classification procedure,* a priori* knowledge about the DNA of the parasites.

It is important to mention that the DNA from both parasites and nonparasites such as white blood cells tends to absorb different amounts of the stain employed. However, the pattern generated by the stained DNA of parasites is clearly different from the pattern generated by the stained DNA of white blood cells. Such a difference is recognized by the pattern recognition algorithms.

### 3.1. Stage 1: Image Acquisition

A group of mice were infected with an inoculation of 5 × 104 blood trypomastigotes of* T. cruzi* via intraperitoneal. Once the mice were infected, the parasitaemia detection started in average between 11 and 15 days afterwards. At this time, the blood smears were prepared and stained using Wright stain, which allows the observation of the morphology of different blood cells, as well as parasites such as* T. Cruzi*,* Leishmania* sp., and* Plasmodium* sp,. After the staining process the blood smears were placed vertically and were left to dry. Finally, an optical Nikon Eclipse E600 microscope was used to take images, first at 10x and then at 100x, with a resulting size increase of 1000 times.

### 3.2. Stage 2: RGB to Grayscale Format

The conversion from an RGB image to a grayscale image involves a simple manipulation of matrices and is performed to reduce the amount of information involved in the learning and detection process. The RGB color model is an additive color model in which red, green, and blue light are added together in different proportions to produce a wide array of colors. A color in the RGB color model is described by indicating how much of each of the red, green, and blue is included. The color is expressed as a triplet (*r*, *g*, *b*) where each component can vary from zero to a defined maximum value, which in our case was 256. RGB images are stored in memory as *w* × *h* × 3 matrices, where *w* is the width and *h* is the height of the image measured in number of pixels. The third dimension of the matrix which is of size 3 corresponds to the 3 different color components of the image: red, green, and blue. To convert any RGB image to a grayscale representation of its luminance, first one must obtain the values of its red, green, and blue components. Then, we need to add together 30% of the red value, 59% of the green value, and 11% of the blue value. These percentages are regarded as typical values. The resulting image is stored in a *w* × *h* image containing pixels of different intensities of the gray color.

### 3.3. Stage 3: Parasites Detection

The Chagas detection process employs a set of Haar-like features and one AdaBoost binary classifier. Haar-like features provide information about clear and dark regions in images. Such information is very useful to detect types of objects that share the same morphological pattern. Since Chagas parasites can be considered objects that share the same shrimp-like shape, they can be detected using the appropriate set of Haar-like features.

**Figure 4:**
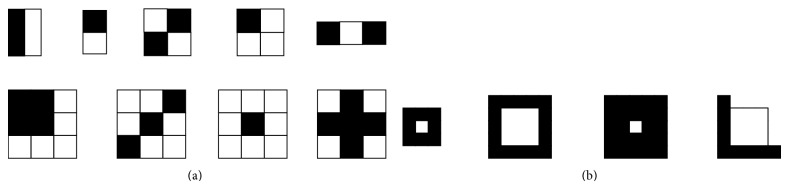
(a) Generic Haar-like features inspired by those proposed by Viola and Jones [7]; (b) Haar-like features specially designed to capture Chagas parasite's morphology.

Given a detection window taken from a specific location of the grayscale image, a Haar-like feature considers adjacent rectangular regions, sums up the pixel intensities contained in each rectangular region and calculates the difference between these sums. This difference is then compared to a learned threshold that separates nonparasites from parasites. The rectangular regions are defined in such a way that relevant dark pixels fall into one same region, meanwhile clear pixels fall into another one.  Figure 4 illustrates the Haar-like features utilized to detect Chagas parasites.

**Figure 5:**
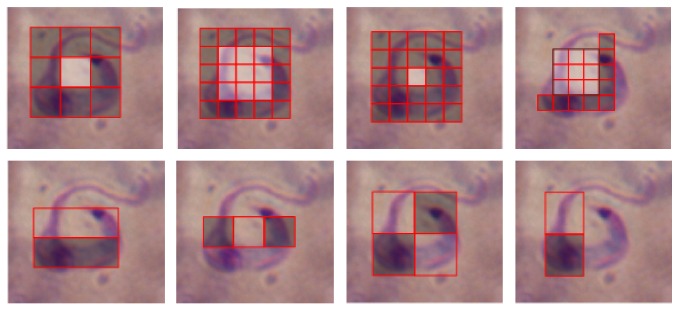
Haar-like features on top can capture more information about the morphology of the Chagas parasite, given their circular shape, than the generic Haar-like features proposed by Viola and Jones, shown on the bottom.

The nine features in Figure 4(a) were inspired by the original Haar-like features proposed by Viola and Jones in [7]. The set of four Haar-like features in Figure 4(b) were designed specifically to represent the shrimp-like shape adopted by Chagas parasites. After running ten times the AdaBoost algorithm and examining the number of times that the algorithm chose the Haar-like features as weak learners, it was possible to effectively discard those Haar-like features that failed to capture important patterns of the Chagas parasites, while keeping only the best ones.  Figure 5 illustrates how specially designed Haar-like features adapt better to the morphology of the parasite.

Moreover, the position of these rectangular regions is defined relative to a detection window that acts like a bounding box to the target parasite. In the detection phase, a window of the target size is moved over the input image, and for each subsection of the image, the Haar-like feature is computed.

The Haar-like features can be computed very fast using the concept of integral image. Given an image *I* of size *R* × *C*, where *R* is the number of rows and *C* is the number of columns, the integral image of a point located in row *r* and column *c* is defined as

(4) $$\begin{array}{c} ii\left(r,c\right)=\underset{r^{\prime }\leq r,c^{\prime }\leq c}{\sum }I\left(r^{\prime },c^{\prime }\right), \end{array}$$

where *I*(*r*′, *c*′) is the gray intensity value of pixels located in row *r*′ and column *c*′. Note that the number of rows increases in the image from top to bottom and the number of columns increases from left to right.

**Figure 6:**
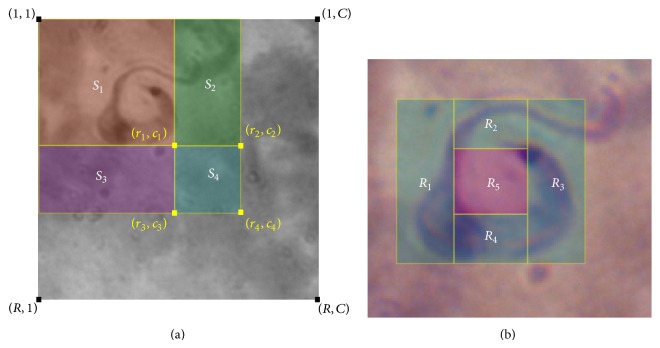
(a) Generic integral image computation; (b) computation of one Chagas-specific integral image.

Now, given the same image *I* and four points *p*_1_(*r*_1_, *c*_1_), *p*_2_(*r*_2_, *c*_2_), *p*_3_(*r*_3_, *c*_3_), and *p*_4_(*r*_4_, *c*_4_) as illustrated in Figure 6(a), and using the definition of integral image of (4), we can compute the regions *S*_1_, *S*_2_, *S*_3_, and *S*_4_ as follows. To calculate the sum of pixels in subwindow *S*_1_, denoted by *σ*(*S*_1_), we simply compute

(5) $$\begin{array}{c} \sigma \left(S_{1}\right)=ii\left(p_{1}\right)=ii\left(r_{1},c_{1}\right). \end{array}$$

For *S*_2_ and *S*_3_ we compute, respectively,

(6) $$\begin{array}{c} \sigma \left(S_{2}\right)=ii\left(r_{2},c_{2}\right)-ii\left(r_{1},c_{1}\right),\\ \sigma \left(S_{3}\right)=ii\left(r_{3},c_{3}\right)-ii\left(r_{1},c_{1}\right). \end{array}$$

Finally, for *S*_4_, we calculate

(7) $$\begin{array}{c} \sigma \left(S_{4}\right)=ii\left(r_{4},c_{4}\right)-ii\left(r_{3},c_{3}\right)-ii\left(r_{2},c_{2}\right)+ii\left(r_{1},c_{1}\right). \end{array}$$

In general, for an image of size *R* × *C*, the whole integral image *ii*(*R*, *C*) can be computed in only one pass over the image and can be stored in a 2-dimensional array. Therefore, to compute a subwindow like *S*_4_ in Figure 6(a), we only need to access the integral image array four times. Furthermore, to compute the integral image of our Chagas Haar-like features, we just need to segment each Haar-like feature in the minimal number *n*_*r*_ of subwindows required to cover it, this number being *n*_*r*_ = 3 for the simplest one and *n*_*r*_ = 5 for the most complex. The computation of one Chagas-specific integral image with 5 subwindows is illustrated in Figure 6(b), where the Haar-like feature template has been divided into 5 regions, namely, *R*_1_,…, *R*_5_ and in order to compute the feature value we need to calculate *σ*(*R*_1_) + *σ*(*R*_2_) + *σ*(*R*_3_) + *σ*(*R*_4_) − *σ*(*R*_5_). In conclusion, the computation of the integral image for our specially designed Haar-like features is still very fast.

### 3.4. Stage 4: Postprocessing

The cascade application of the AdaBoost algorithm together with the appropriate set of Haar-like features is highly effective in finding most of the true Chagas parasites. However, it also detects a small number of false parasites. The postprocessing filter described in this section helps to discard false parasites and decreases the false positive rate of our method.

**Figure 7:**
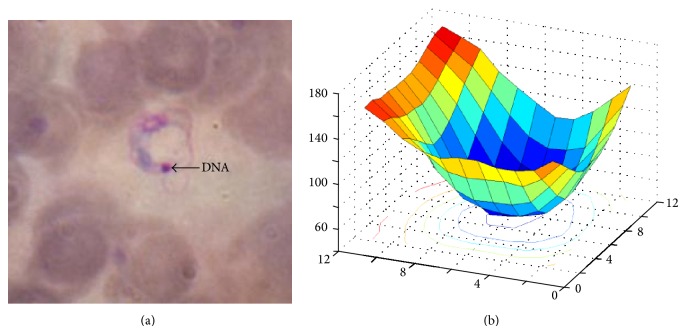
(a) Dark spot of pixels generated by an accumulation of DNA; (b) a DNA spot seen as a surface of 11 × 11 pixels.

The postprocessing filter is focused on the analysis of one dark spot of pixels appearing in all parasites' bodies. This spot, indicated by an arrow in Figure 7(a), corresponds to an accumulation of DNA of the Chagas parasite. When the image is plotted as a surface, it can be seen that such DNA spot has one particular valley-like shape, shown in Figure 7(b). Since the shape of this dark spot and the low intensity values taken by their corresponing pixels create a pattern very useful to discriminate between parasites and nonparasites, they were used as a source for feature extraction as it is described next.

Given that the stained pixels have low intensities, these values being not greater than 80, in the green component of the RGB image, the following three features were used to train a SVM.

*(i) Feature 1*. Given a subwindow detected by AdaBoost, the percentage of pixels that have intensities at most 80 was computed. This percentage, which represents the size of the stained region of the parasite, is the first feature.

*(ii) Feature 2*. The mean of the intensities of all pixels with individual intensities at most 80 was encountered in the subwindow detected by AdaBoost.

*(iii) Feature 3*. The standard deviation of the intensities of all pixels with individual intensities at most 80 was encountered in the subwindow detected by AdaBoost.

The choice of the green component over the red and the blue ones was decided after a careful visual examination of a subset of images in the original RGB version and in all 3 individual components: red, green, and blue. After such visual examination, it was clear that the green component was superior to the others in terms of discriminative information. In other words, the information provided by the green channel allows us to perform a more accurate classification of parasites and nonparasites.

## 4. Experimental Work

### 4.1. Experimental Methodology

We tested the proposed algorithm using a data collected in the Instituto de Investigaciones Regionales at the Universidad Autónoma de Yucatán, México. We had available for our study a total of 120 color images of dimension 256 × 256 pixels. Sixty of these images were specially selected to contain a Chagas parasite. Meanwhile, the other 60 remaining images were selected to contain nonparasites. Machine learning was employed to generate our basic classifier algorithm, which was then used for the more general Chagas parasite detection process. The results we provide in this paper were obtained using a typical pattern recognition methodology [26], using training and testing sets of images.

A 10-fold cross-validation procedure was used for training and testing the AdaBoost and the SVM + Feature Extraction learning methods. Since AdaBoost is sensible to rotation, during the training phase, we generated a total of 840 positive examples of parasites images by rotating the original images in increasing amounts of 15 degrees. For every experiment, we used 756 positive images for training, leaving 84 positive images for testing. Even though we were limited by the number of positive examples available, the number of negative examples could be defined as desired. All negative examples were generated by random sampling from the original 256 × 256 pixels images. For training, AdaBoost used as many negative examples as possible to reduce the false positive rate, and the final learned model was tested with 10800 negative images. In the case of the SVM + Feature Extraction methods, we used 1296 negative images for training and 10800 for testing. It is worth mentioning that once the classifiers have been trained, the proper analysis of a 256 × 256 image, which means that the algorithm is actually looking for parasites, is performed in real time. We are talking about a few milliseconds, using a commercial laptop or desktop computer. Therefore, several hundred images can be analyzed in a matter of minutes. Moreover, our program was implemented in Matlab using 2 public libraries: fdtool, for the AdaBoost implementation, and libsvm, for the support vector machine implementation.

#### 4.1.1. Comparison with Support Vector Machines

The application of SVM learning requires the computation of one feature vector for each subwindow that needs to be classified as parasite or nonparasite. Such features vectors are then passed to the SVM classifier which makes the final decision. The selection of the features is a key part of the classification process, simply because by using the wrong features the learning algorithm cannot extract the patterns required to build a confident classifier, resulting in a high classification error. In order to obtain the best results with the application of SVMs, we reviewed the most relevant published works involving SVM learning and malaria parasite detection. So that we can use for our experimental work those features that have been previously reported to provide high quality results.

Color, shape, and texture features were selected to form a 38-dimensional feature vector. The color and shape features are computed from 7 different histograms that contain information about the red channel and green channel of the RGB image, the hue and saturation component from the HSV image, the grayscale pixel intensities and the result of the application of the sobel operator horizontally and vertically over the RGB image. Once the 7 histograms are computed, 5 values of each histrogram form the first 35 features. The values taken from the histograms are the mean, the standard deviation, kurtosis, skewness, and entropy. Finally, 3 more features known as Tamura texture parameters are computed from the original RGB images. Those final parameters are coarseness, contrast, and direction. All theses features are classified in [15] as part of their work in automatic detection of Malaria parasites and they are described in [27].

#### 4.1.2. Sensitivity, Specificity, and *F*-Measure

In order to evaluate the performance of our implemented AdaBoost classifier, we used three different statistical metrics: sensitivity, specificity, and effectiveness. Sensitivity is the probability of a positive test given that the patient is ill and it is computed as follows:

(8) sensitivity =number  of  true  positivesnumber  of  true  positives+number  of  false  negatives.

Specificity in turn is the probability of a negative test given that the patient is well and it is computed as follows:

(9) specificity =number  of  true  negativesnumber  of  true  negatives+number  of  false  positives.

Due to the unbalanced class distribution between subwindows containing a Chagas parasite and subwindows containing something else, the use of statistical metrics related to the effectiveness were also considered. In these experiments, the *F*-measure is used:

(10) $$\begin{array}{c} F_{\beta }=\frac{\text{TPR}*\text{PR}}{\beta *\text{TPR}+\left(1-\beta \right)*\text{PR}} \end{array}$$

with

(11) $$\begin{array}{c} \text{TPR}=\frac{\text{TP}}{\text{TP}+\text{FN}},\\ \text{PR}=\frac{\text{TP}}{\text{TP}+\text{FP}}, \end{array}$$

where TP stands for the true positives, FN for the false negatives, and FP for the false positives. The *β* coefficient (0 < *β* < 1) allows to assign relative weights to both the true positive and precision rates. In these experiments, *β* = 0.05 is used, so the search was addressed to detection of TP.

### 4.2. Experimental Results

#### 4.2.1. AdaBoost Results

**Figure 8:**
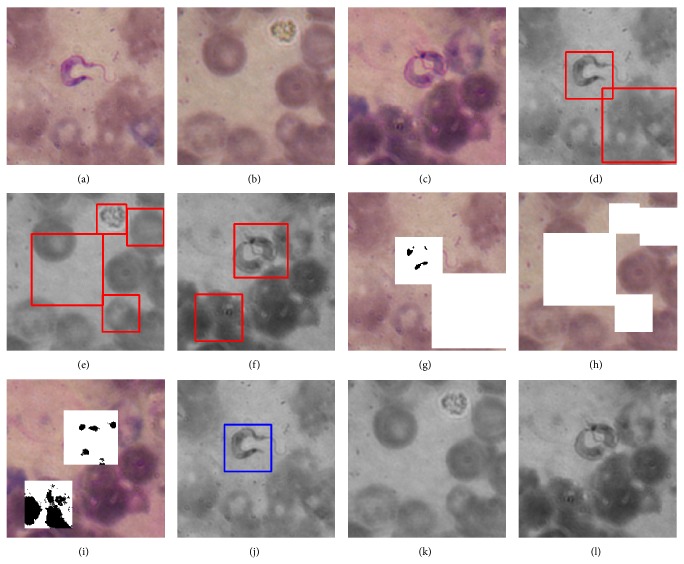
Results of the Chagas parasite detection algorithm with a sample of images. Each column corresponds to a different example of detection. The first row contains the color images in RGB format; the second row shows the grayscale images once the parasites have been detected by the classifier; in the third row we show the result of the postprocessing stage; and the fourth row shows the final result of the detection systems.

**Figure 9:**
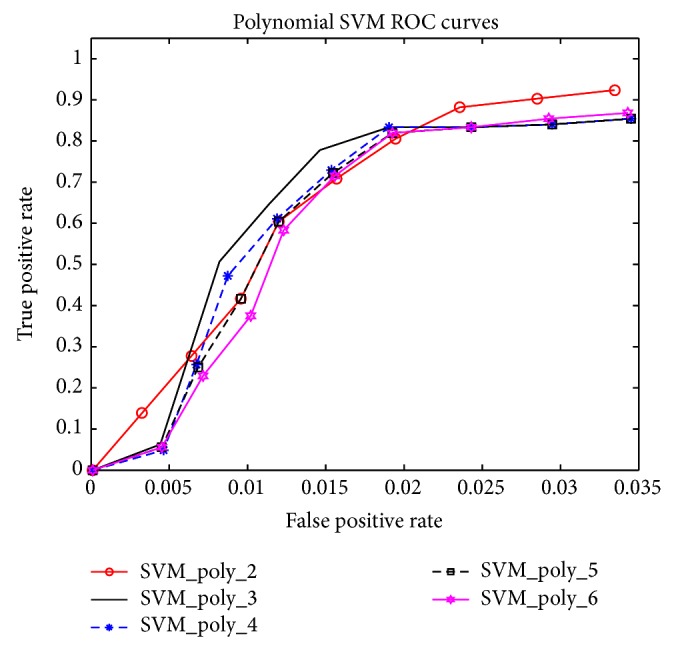
ROC curves with SVMs and polynomial kernels ranging from degrees 2 to 6.

**Figure 10:**
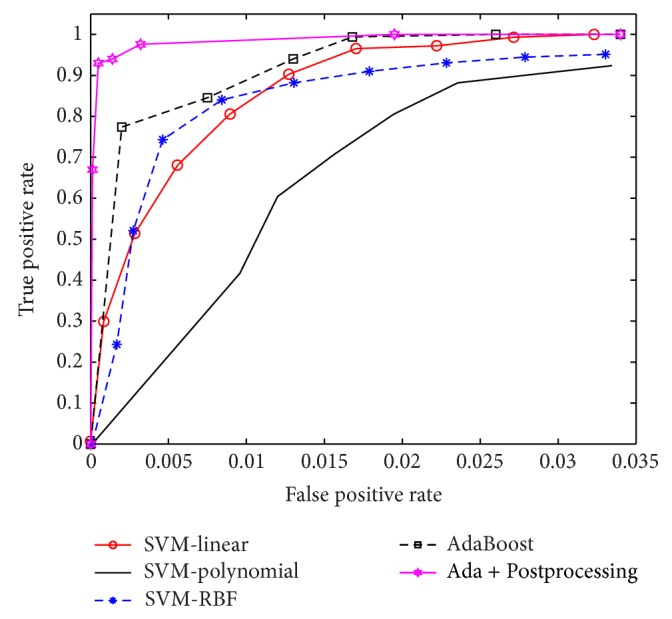
ROC curves using AdaBoost and SVMs with three different types of kernel: linear, polynomial, and RBFs.

Looking at the values provided in Table 2, we see that AdaBoost methods are as good as SVM methods in terms of sensitivity, with mean value of 1 and standard deviation of 0. All five methods were trained to detect every parasite in the test sets. In terms of specificity, AdaBoost + Postprocessing is clearly the winner among the five methods being compared, with mean value of 0.9325 and standard deviation of 0.0496, as given in Table 3. Tables 4 and 5 also show that AdaBoost + Postprocessing is superior to the others, in terms of *F*-measures. In Figure 8, we illustrate the result of our AdaBoost algorithm for Chagas parasite detection. These results were obtained with a cascade of weak classifiers of 11 stages.  Figure 9 provides the ROC curves for SVMs using different degrees of polynomial kernels and Figure 10 compares the ROC curves for AdaBoost and SVMs methods using different types of kernels.

#### 4.2.2. SVM + Feature Extraction Results

SVM results are good especially using linear and polynomial kernels. Features used to train the SVM proved to contain the needed information to build good classifiers. Those features were created from color histograms and some other values that encapsulate texture information of the image. The best results for each type of kernel were obtained with the library libsvm, finding first the best pair of parameters *γ* and *C* for our training examples. Those parameters are given in Table 1. The AdaBoost + Postprocessing method was implemented using a linear kernel SVM with parameters *γ* = 0.002 and *C* = 0.5. These parameters were obtained through multiple cross-validation experiments using different pairs of values for the *γ* and *C* parameters.

#### 4.2.3. AdaBoost versus SVM + Feature Extraction Comparison

Results obtained with AdaBoost are more robust than those obtained with SVM. However, the sensitivity and specificity reached by using SVM cannot be considered bad, given the difficulty of the task and their performance is comparable to the performance obtained in the malaria parasite detection task.

## 5. Discussion

Based on the results obtained with both machine learning methods, AdaBoost and SVM, we can see that taking into account features related to shape, color and texture seems to be the most robust way to go when we target a parasite detection task. The main advantage in using AdaBoost with the approach introduced by Viola and Jones is the fast computation of the features using the integral images trick, as opposed to any other method of image segmentation that are commonly used together with the SVM approach. Image segmentation is a time-consuming task and its use without parallel computation is almost impossible for applications that require to scan many images in a short time.

The consideration of DNA pixels through a trained SVM in the Ada + Postprocessing method is handled as a postprocessing procedure because it is also a time-consuming procedure which should be applied a reduced number of times. If we attempt to interwine this counting procedure with the typical AdaBoost algorithm using integral images, we simply nullify the major advantage of the integral image trick, which is a fast computation of the pixels addition.

**Figure 11:**
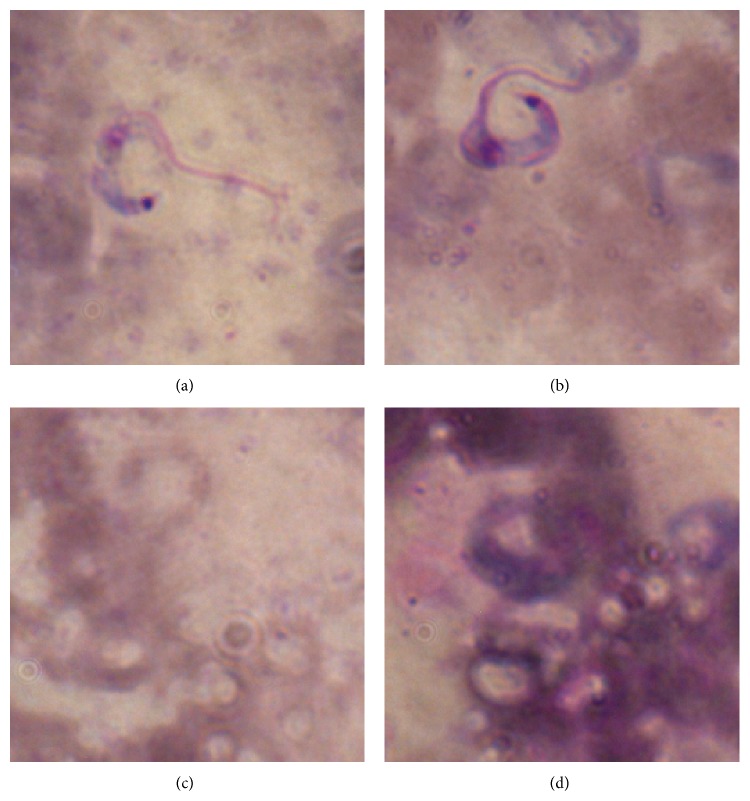
Two representative images. In image (a), the number of dark pixels makes it easy for the detection system to consider it as a valid parasite. In image (b), the number of dark pixels is larger than average, which makes it difficult for the detection system to consider it as a valid parasite. An easy background looks like (c) and a difficult one looks like (d).

In Figure 11, we present some examples of images that are representative cases of ease and difficulty of classification. When an image contains a high percentage of pixels stained in dark purple, as in Figure 11(d), the algorithm finds it more difficult to discard false positives.

Parasites and other stained objects like blood cells are nonrigid and therefore vary in shape and size. The color is important but its only use to distinguish between the Chagas parasites and other things is not enough. On the other hand, raw images cannot be used directly as a feature vector for two main reasons: first the size of such a vector would increase unnecesarily the time of computation and second the high variation in shape and size would make it extremely difficult for any machine learning algorithm to learn the correct classifier. In the ideal case, features must be able to capture shape and color characteristics of the objects to be classified.

Failing to detect Chagas is certainly a more serious situation than having a false positive error. Diagnosing a person suffering from Chagas as not having the disease would put her life at risk. A false positive alarm on the contrary would only incur in a labor cost for the doctor in charge of confirming whether the parasite is present in the blood sample.

In practice, the detection of* T. cruzi* by analysis of digital images obtained from peripheral blood smears would bring multiple benefits associated to the Chagas disease diagnosis. First, it would make possible the diagnosis of the disease in its initial phase, which is the acute phase, because the presence of the parasite in the blood would indicate with high probability that the infection is present. As a consequence of the early diagnosis, the human cases could be treated with drugs such as Nifurtimox or Benznidazole, and the probability of stopping the disease is very high. Second, given that human cases of Chagas are very difficult to be diagnosed during the analysis routinely performed in national laboratories in countries such as Mexico, an automated Chagas detection system would contribute to estimate the local impact of the disease and to determine the existence of any endemic region.

Moreover, the successful implementation of this algorithm, would motivate the laboratories and hospitals to reconsider the usefulness of the blood smears analysis and to trust this test as a confident laboratory diagnosis. Furthermore, the laboratory technicians would reduce the number of hours they need to sit in front of a microscope to analyze so many samples day after day. Situation that is currently originating them problems such as visual fatigue and frequent pain in the back.

Finally, this algorithm can be integrated as part of one automated microscopic system for the adquisition of images from blood smears. Such a device would be composed of one optical microscope with a controllable electromechanical surface specially designed to move the blood smears as needed by the scanning software. The microscope would be equipped with a high-resolution camera in order to take the images. Images would be stored in hard drives to allow their posterior analysis with our Chagas detection algorithm. This device is currently being built as part of one CONACYT research project on Chagas led by Dr. Ruiz-Piña in Mexico (project code: salud-2009-01-113848).

## 6. Conclusions

In this paper, we have provided an approach to the Chagas parasite detection problem based on AdaBoost learning. Using the approach used by Viola and Jones for the task of face detection, we obtained high sensitivity and specificity values. Chagas detection approaches based on machine learning and computer vision methods have been barely studied as it is evidenced by the lack of literature on the topic. The most promising method proposed for the detection of malaria parasites was implemented and compared to AdaBoost. This method consists basically in the use of different kinds of values taken from color histograms in order to form the feature vector that are later used to train a support vector machine. Both shape and color proved to be important in the task of Chagas parasite detection when high level of sensitivity and specificity are required. Applying SVM for the task of Chagas parasite detection requires the computation of the 38-dimensional feature vector for each subwindow that we want to classify as being a parasite or nonparasite. However, if we proceed in this way with every subwindow of the original image of size 256 × 256, the whole process would be highly time-comsuming. One way to reduce the number of subwindows that are worth checking is by means of segmentation. Segmentation allows us to identify different relevant objects in the image and discard those that does not look like parasites. Given that most of the objects detected during the segmentation are discarded, only a minor number of subwindows are further check by the SVM. Although the use of segmentation is very common for this kind of tasks, it can also be a time-consuming process. AdaBoost applied with integral images does not require segmentation, because every subwindow can be checked in constant time, regardless of its size.

## Figures and Tables

**Table 1:** Best *γ* and *C* parameters for SVMs according to the type of kernel employed and our training data.

Kernel	*γ*	*C*
Linear	0.00003	8
Polynomial	0.0078	0.0312
Radial basis function	0.00003	8192

**Table 2:** Sensitivity by method.

Method	Mean	Std. Dev.
AdaBoost	1	0
AdaBoost + Postprocessing	1	0
SVM linear + Feature Extraction	1	0
SVM polynomial + Feature Extraction	1	0
SVM RBF + Feature Extraction	1	0

**Table 3:** Specificity by method.

Method	Mean	Std. Dev.
AdaBoost	0.7550	0.1820
AdaBoost + Postprocessing	0.9325	0.0496
SVM linear + Feature Extraction	0.8218	0.0118
SVM polynomial + Feature Extraction	0.6691	0.0035
SVM RBF + Feature Extraction	0.7111	0.0068

**Table 4:** *F*-measure (*β* = 1) by method.

Method	Mean	Std. Dev.
AdaBoost	0.7550	0.1820
AdaBoost + Postprocessing	0.9325	0.0496
SVM linear + Feature Extraction	0.8218	0.0118
SVM polynomial + Feature Extraction	0.6691	0.0035
SVM RBF + Feature Extraction	0.7111	0.0068

**Table 5:** *F*-measure (*β* = 10) by method.

Method	Mean	Std. Dev.
AdaBoost	0.9976	0.0018
AdaBoost + Postprocessing	0.9993	0.0004
SVM linear + Feature Extraction	0.9982	0.0001
SVM polynomial + Feature Extraction	0.9967	0.0000
SVM RBF + Feature Extraction	0.9971	0.0001
